# Risk factors for mild depression in older women with overactive bladder syndrome—A cross sectional study

**DOI:** 10.1371/journal.pone.0227415

**Published:** 2020-01-30

**Authors:** Raquel H. Jacomo, Aline T. Alves, Patrícia A. Garcia, Marianne L. da Silva, Liana B. G. Matheus, Dayanne C. R. Lorena, João B. de Sousa

**Affiliations:** 1 Medicine School- University of Brasilia, Brasília, Distrito Federal, Brazil; 2 Department of Physical Therapy, University of Brasilia, Brasília, Distrito Federal, Brazil; 3 Department of Physical Therapy, Federal University of Goias – Jataí, Goiás, Brazil; University of Insubria, ITALY

## Abstract

**Background:**

Studies demonstrate an association between severe depression and overactive bladder syndrome (OAB). However, mild depression is constantly overlooked. The aim of this study was to evaluate the clinical and sociodemographic factors associated with mild depression in women with OAB.

**Methods:**

Cross-sectional study involving 241 women over 60 years old in Brasilia, Brazil. All patients were subjected to an interview followed by questionnaires and physical examination. The clinical and sociodemographic variables analyzed were age, body mass index, physical activity level, OAB symptoms, presence of gynecological surgery, fecal incontinence, systemic arterial hypertension, Diabetes Mellitus, anxiety (Beck Anxiety Scale). The Geriatric Depression Scale-15 (GDS-15) was used to identify depression. Univariate logistic regression was used to assess the correlation between mild depression and the variables chosen. Variables with a p-value less than 0.2 were included in the multivariate logistic regression analysis. The level of confidence was set at 95%.

**Results:**

121 volunteers suffered from mild depression. The multivariate analysis demonstrated that gynecological surgery (p < .001) and anxiety (p < .001) are factors associated with mild depression. Older women with a history of gynecological surgery and a GDS-15 score of 2.04 were 1.08 times more likely to develop mild depression compared to older women with no history of gynecological surgery.

**Conclusion:**

Anxiety and a history of gynecological surgery are factors that need to be taken into account and may influence the development of mild depression in older women with OAB. Psychological treatment should be considered an important adjunct in the treatment of women with symptoms of Overactive Bladder Syndrome.

## Introduction

Overactive bladder syndrome (OAB) is a common condition characterized by urgency to void, usually accompanied by frequency and nocturia, with or without urge-urinary incontinence (UUI), in the absence of urinary tract infection (UTI) or an underlying metabolic or pathologic condition [1]. It is estimated that 16–25% of women have some type of lower urinary tract symptom (LUTS), mainly OAB [2,3]. OAB symptoms increase with advancing age in men and women. In the United States, approximately 35% of women aged 75 or older were found to have OAB [4]. The impact of OAB is broad and significant, affecting the quality of life of patients in general, as well as their social, emotional, psychological and sexual lives [5,6].

Depression is one of the most common and widespread mental illnesses in the world, and a major contributor to the global burden of disease [7]. End-of-life depression is a public health problem which is associated with an increased risk of morbidity and suicide. In addition, it is associated with physical and cognitive decline, reduced social life, and greater self-neglect, thereby increasing mortality. [8]. The prevalence of depressive symptoms in the population over 60 years old may reach 40% [9]. In older people, depression signs can be highly heterogeneous, and often not noticeable, leading to a high risk of late intervention [10]. One study shows that 38.3% of postmenopausal women have mild depression [11].

The relationship between severe depression and overactive bladder symptoms is well established. People with OAB Syndrome are at a greater risk of developing depression than people without the condition. A study controlling for age as a confounding variable found that patients with depression have worse OAB symptoms, and their quality of life is more seriously affected compared to those without depression [6]. Similarly, Melotti et al. showed a correlation between severe depression and OAB symptoms [12]. In a study with 2,877 women aged 65 years or older, Sexton et al. found that depression was an important factor to the worsening of OAB symptoms [13].

However, none of the studies cited conducted a detailed analysis regarding mild depression. In fact, we do not know if there is any association between OAB symptoms and mild depression and what factors are at play. There is a gap in the literature: Is there a correlation between mild depression and overactive bladder symptoms? Could public policies be more effective? And, consequently, could there be better preventive measures to avoid mild depression evolving into severe depression? Risk and protective factors contribute more or less to the etiology of depression depending on their frequency and importance over the course of life [14]. Establishing whether these factors are associated with overactive bladder requires better-informed clinical management, good diagnostic practices and personalized interventions aimed at improving the quality of life of elderly patients.

The aim of this study was to evaluate the clinical and sociodemographic factors associated with mild depression in older women with OAB.

## Methods

A cross-sectional study was conducted. We recruited 260 consecutive women with a clinical diagnosis of OAB from the Program for Micturition Dysfunction at the Brasília Health Center (Brazil), from October 2013 to October 2018. The study was approved by the Research Ethics Committee of the Faculty of Health Sciences of the University of Brasília (CAEE-089707.13.8.00000.0030- September, 30 2013). All the patients signed an Informed Consent Form.

Participants were eligible for inclusion in the study if they were between 60–80 years of age and had a clinical diagnosis of OAB as defined by the ICS (International Continence Society) [1]. The exclusion criteria were patients with positive urinalysis and urine culture, patients who had been exposed to irradiation or hormonal therapy in the previous six months, patients with neurological diseases, patients using anticholinergic drugs, calcium antagonists, b-antagonists, or dopamine antagonists, patients with a grading ≥ grade III according to the pelvic organ prolapse quantification system (POP-Q), and those unable to satisfactorily answer the questionnaires and with severe depression.

The patients selected were interviewed by a healthcare professional specialized in micturition disorders to obtain the clinical and sociodemographic variables. Possible factors included age, BMI (continuous variable), level of physical activity (active or non-active), fecal incontinence, history of gynecological surgery, systemic arterial hypertension (SAH), Diabetes Mellitus (DM), and presence of anxiety. For the evaluation of depressive symptoms, we used the Geriatric Depression Scale-15 (GDS-15), a scale that had been previously validated to screen mood disorders in older adults. The GDS-15 score as an instrument for screening depression was defined as follows: a score of 0–5 indicated individuals without depression, 6–10 suggested mild depression, and 11–15 revealed severe depression [15,16]. Anxiety was assessed by using the Beck Anxiety Inventory (BAI). The BAI is a previously validated questionnaire, consisting of 21 items that evaluate different anxiety symptoms. Based on the individual's own assessment and perception in the last 4 weeks, the symptoms are classified as minimal when the score varies from 0–10; mild, when it varies between 11–19; moderate, between 20–30; and severe, between 31–63 [17]. To assess the symptoms of overactive bladder, the International Consultation on Incontinence Questionnaire (ICIQ-OAB), recommended by the ICS, was used, and the higher the score, the greater the symptoms of OAB [1].

The cutoff values adopted to assess nutritional status, as well as the presence of SAH and DM were in line with the recommendations by the World Health Organization (WHO) [18,19]. Based on a nutritional study using BMI, older adults were classified as follows: underweight when BMI ≤ 18.5 kg/m^2^; normal weight when 18.5 kg/m^2^ < BMI <25 kg/m^2^; overweight when 25 kg/m^2^≤ BMI <30 kg/m^2^), and obese when BMI ≥ 30 kg/m^2^ [20].

Volunteers who were physically inactive were considered sedentary, whereas participants who did physical activities at least twice a week and participated in the exercise programs offered by the primary care network were considered physically active. Gynecological surgery refers to surgery on the female reproductive system. This includes procedures for benign conditions, cancer, infertility, and incontinence [21].

### Data analysis

We performed a sample calculation using the G Power 3.1.9.2 program with data obtained from a pilot study of 15 elderly women without depressive symptoms and 15 with mild depressive symptoms. Including the independent variables separately, i.e., fecal incontinence and gynecological surgery, and using the presence or absence of symptoms of mild depression as dependent variables for the univariate logistic regression, and considering a power of 90% and an alpha error of 0.05, a sample of 229 participants was determined. We added 5% more to account for data loss, totaling 241 participants.

The non-normal distribution of the data was confirmed by the Kolmogorov-Smirnov test. The categorical data were presented in terms of frequency and percentage. Non-normally distributed continuous data were presented as median and interquartile range (25–75%). Continuous data were compared between the two groups of older women using the Mann-Whitney U test (mild depression and no-mild depression). Categorical data were compared using the Chi-square test. Univariate logistic regression analysis was used to determine the association between each independent variable and the dependent variable (symptoms of depression).

The factors included in the model were age, BMI (continuous variable), physical activity level (active or non-active), fecal incontinence, history of gynecological surgery, SAH, DM, and presence of anxiety (continuous variable). *Odds ratios* (OR) were calculated for each explanatory variable with 95% confidence intervals. Subsequently, variables that showed a p-value of association of less than 0.20 in the univariate regression analysis were included in the multivariate logistic regression analysis [20]. The best model was selected based on the procedure of reduction of variables, using the *enter* method. The significance level considered was 0.05. Data analysis was performed using the *Statistical Package for Social Sciences* (SPSS), version 16.0. The null hypothesis was rejected for P-values <0.05.

## Results

This study involved 260 women with a clinical diagnosis of OAB. Nineteen patients failed to meet the inclusion criteria, 16 of them had severe depression and 3 were unable to answer the questionnaires. Thus, data from 241 patients were analyzed. It was observed that 50% of the patients had no symptoms of depression and 50% had mild depression. The mean age was 67 (64–73) years old. In the group studied, 107 (23.2%) patients were married, 135 (56%) did some type of physical activity, 117 (48.5%) did not finish elementary school, and 87 (37.3%) were obese. As for the obstetric history, the median was five pregnancies and three vaginal deliveries. There were no differences between groups regarding clinical and sociodemographic characteristics. Eighty-six (35.7%) patients had no or low anxiety, 75 (31.1%) had mild anxiety, 61 (25.3%) had moderate anxiety and 19 (7.9%), severe anxiety (Table 1).

**Table 1:** Characteristics of older women with OAB. Frequency (percentage). Median [interquartile 25–75%]. Mann-Whitney U test for continuous data. Chi-square test for categorical data. p<0,05.

Characteristics	Total Sample	Older women without depression	Older Women With mild depression	p-value
**Age (years)**	67 [64–73]	67 [64–73.5]	67 [64–73]	0.597
**Marital status (%)**				
Single	56 (23.2)	34 (24.5)	22 (21.6)	0.598
Married	107 (44.4)	61 (43.9)	46 (45.1)	
Divorced	2 (0.8)	2 (1.4)	0 (0.0)	
Widow	76 (31.5)	42 (30.2)	34 (33.3)	
**Level of Education**				
illiterate	36 (14.9)	20 (14.4)	16 (15.7)	0.570
incomplete elementary schooll	117 (48.5)	66 (47.5)	51 (50.0)	
complete elementary school	53 (22.0)	34 (24.5)	19 (18.6)	
Incomplete high school	29 (12.0)	15 (10.8)	14 (13.7)	
complete high school	1 (0.4)	4 (2.9)	1 (1.0)	
graduate	5 (2.1)		1 (1.0)	
**BMI** (Kg/m^2^)	28.3[24.6–31.4]	28.8[24.6–30.7]	28.7[24.9–34.4]	0.091
Underweight <18.5	3.(1.3)	2(1.5)	1(1.0)	
Normal Weight 18.5–24.9	61 (26.2)	38 (28.4)	23 (23.2)	0.395
Overweight 25–29.9	87 (37.3)	53(39.6)	34 (34.3)	
Obese ≥ 30	82(35.2)	41 (30.6)	41 (41.4)	
**Gestations**	5 [3–7]	4 [3–7]	5 [3–7]	0.089
**Vaginal birth**	3 [2–5]	3 [2–5]	3 [2–5]	0.794
**Fecal incontinence**				
Continent	189 (79.7)	115 (83.9)	74 (74.0)	0.072
Incontinent	48 (20.3)	22 (16.1)	26 (26.0)	
**Urogynecological Surgery**				
No	120 (49.8)	62 (44.6)	58 (56.9)	0.068
Yes	121 (50.2)	77 (55.4)	44 (43.1)	
**Physical Activity Level**				
Inactive	106 (44)	61 (43.9)	45 (44.1)	0.538
Active	135 (56)	78 (56.1)	57 (55.9)	
**SAH**				
No	64 (26.6)	41 (29.5)	23 (22.5)	0.241
Yes	177 (73.4)	98 (70.5)	79 (77.5)	
**DM**				
No	182 (75.5)	104 (74.8)	78 (76.5)	0.880
Yes	59 (24.5)	35 (25.2)	24 (23.5)	
**Anxiety (score)**	14 [8–22]	11 [6–19]	20 [12–26] 16	<0.00
Minimum	86 (35.7)	70 (50.4)	(15.7)	1
Medium	75 (31.1)	40 (28.8)	35 (34.3)	
Moderate	61 (25.3)	23 (16.5)	38 (37.3)	<0.00
Severe	19 (7.9)	6 (4.3)	13 (12.7)	1
**Symptoms of OAB (ICIQ-OAB)**	9 [7–11]	9 [6.5–11]	9 [7–11]	0.367

The univariate logistic regression analysis showed that BMI, fecal incontinence, history of gynecological surgery and anxiety were factors associated with mild depression in OAB patients (Table 2).

**Table 2:** Univariate and multivariate logistic regression analysis to identify factors associated with mild depression in older women with overactive bladder syndrome (n = 241). Variables with p-value <0.20 in the univariate analysis were included in the multivariate regression analysis. **p < .20 Multivariate binary logistic regression with *enter* method.^†^p<0.05.

Risk Factors	Univariate Analysis		Multivariate analysis	
	OR	95% CI	OR	95% CI
Age (Years)	0.983	0.946–1.023	-	-
BMI (kg/m^2^)	1.049**	0.999–1.101	1.033	0.980–1.089
Level of physical activity	0.991	0.592–1.658	-	-
Fecal incontinence	1.837**	0.970–3.478	0.612	0.305–1.227
Urogynecologic Surgery ^†^	0.611**	0.365–1.022	2.043	1.138–3.666
HAS	1.437	0.796–2.593	-	-
DM	0.914	0.503–1.660	-	-
Anxiety ^†^	1.084**	1.052–1.117	1.080	1.047–1.114
Symptoms of overactive bladder (ICIQ-OAB) ^‡^	1.033	0.957–1.114	-	-

After the multivariate analysis, history of gynecological surgery and anxiety were maintained in the final model as independent variables associated with mild depression (Table 2). These findings reveal that older women with a history of gynecological surgery and a GDS-15 score of 2.04 were 1.08 times more likely to develop mild depression compared to older women with no history of gynecological surgery.

## Discussion

To our knowledge, this is the first study that investigated mild depression and its influence in older women with OAB symptoms. This study demonstrated that history of gynecological surgery and anxiety are factors associated with mild depression in women aged 60–80 with overactive bladder syndrome.

Despite the 20% prevalence in adults, depression often goes undiagnosed, thus often untreated [22].

Untreated depression is associated with an increase in the amount and severity of physical and mental comorbidities such as insomnia, anxiety, and voiding problems [22]. Most studies concluded that severe depression is associated with OAB symptoms. Some authors, for example, examined the connection between LUTS and suicidal ideation in 2,890 men aged 40 or older using data from the National Health and Nutrition Examination Survey (NHANES) [23]. A recent cross-sectional study with 274 young women showed that moderate to severe depression could be found in 59.8% of OAB patients [12].

History of gynecological surgery is a factor that may lead to depression in elderly patients. Nowadays, hysterectomy is the most common gynecological surgery in the world [23,24]. According to several reports from 2012, more than a third of women over 60 in the United States underwent some kind of gynecological surgery [24]. In the present study, 50.2% of the patients also underwent some kind of gynecological surgery. The impact gynecological surgery has on micturition dysfunctions is known, especially hysterectomy, which causes damage to the structures supporting the bladder and urethra. It is believed that 20% of hysterectomy patients may develop OAB symptoms [25].

In addition to OAB symptoms, hysterectomy may be associated with depression and cognitive symptoms, although studies are still conflicting. Brandner et al., 2018, showed, an overall decline in cognitive function in a vulnerable group of elderly women during the short-term postoperative period [26]. Lewicka et al. concluded that women submitted to gynecological surgery were more likely to have depression. Even those who underwent vaginal gynecological surgeries had a higher level of depression compared to those who underwent laparoscopy [25]. However, Bahri et al. found no association between depression and hysterectomy in patients 3 months postoperatively [27]. Both studies evaluated patients in a short period of time. It is unclear if the presence of gynecological surgery could influence depression in the long term. The literature is scarce when evaluating long-term depression in patients who underwent gynecological surgery. Mac Donald et al. concluded that women who undergo hysterectomy for pelvic pain and endometriosis under the age of 30 are more likely to have residual symptoms than older women [28]. Another study, conducted in two Chinese cities, concluded that women who undergo sterilization procedures have 2.34 times more risk of developing depression. These findings suggest a need for careful pre-sterilization counseling and education, as well as the option of psychotherapy for those who have undergone the procedure [29].

The present study demonstrated a high prevalence of patients with anxiety, 64.3%, confirming some previous studies [30,31]. The correlation between anxiety and depression is well established. These results lead us to believe that mild depression is associated with anxiety, and not to OAB symptoms, and perhaps severe depression is associated with worse levels of OAB symptoms. The association between anxiety, depression and bladder function is based on a common neurochemical explanation. A compelling association between central and peripheral/norepinephrine (NE) serotonin systems and lower urinary tract function has been proposed. The studies support that the serotonergic system plays a role in the regulation of anxiety in adolescence and also during adult life. An experimental study in rats showed that reduced levels of serotonin, which is also linked to depression, leads to increased urinary frequency and overactive bladder. Another possible explanation is the deregulation of the hypothalamic-pituitary-adrenal axis, resulting in an increase in the corticotropin releasing factor (CRF) which, in turn, would increase the adrenocorticotropic hormone (ACTH) and cortisol, both associated with depression and hormone-dependent bladder function. Even though biological factors alone are unable to modify the symptoms of OAB in mildly depressed older women, when associated with other factors in women with high levels of OAB symptoms, they could help explain worse levels of depression and anxiety [32–34].

Even though the sample studied was representative, there are some limitations. First, some degree of selection bias, since the older women selected were participants of a wellness program specifically targeting voiding dysfunctions. Second, the present study is transverse in nature. The inherent limitation of a cross-sectional study is that data collection occurs at only one point in time, so it may be difficult to elucidate cause and effect. It would be important to follow this population for a longer period of time. The third limitation of the study was the lack of description of the type of gynecological surgery procedure the patients underwent. Therefore, new studies are needed in order to support the arguments presented.

Clinically, we believe that a preoperative psychological assessment of patients undergoing gynecological surgery is important. A multidisciplinary approach to identify risk factors in patients undergoing surgery is also required. These results should be taken into consideration for preoperative counseling and decision-making purpose. It is also worth noting that the factors studied in the present study have an influence on the depressive symptoms of elderly women with OAB. It is important that future studies explore them, but also other factors that may not have been considered by us.

## Conclusions

Anxiety and history of gynecological surgery are factors associated with mild depression in older women with overactive bladder syndrome. Psychological treatment should be approached as an important adjunct to the treatment of women with symptoms of overactive bladder. This study marks the beginning of a study on mild depression in women with OAB and associated factors. Given the large number of women who undergo annual gynecological surgeries and the high prevalence of anxiety in women with overactive bladder, it is important to have specific public services targeted at preventing the disease and caring for this population.

## List of abbreviations

ACTH: adrenocorticotropic
BAI: Beck Anxiety Inventory
BMI: body mass index
CAEE: Research Ethics Committee of the Faculty of Health Science
CRF: Corticotropin releasing factor
DM: Diabetes Mellitus
GDS-15: geriatric depression scale-15
ICIQ-OAB: International consultation on incontinence- overactive bladder
ICS: International Continence Society
LUTS: low urinary tract symptoms
NE: norepinephrine
NHANES: National Health and Nutrition Examination Survey
OAB: overactive bladder syndrome
POP-Q: Pelvic organ prolapse quantification
SAH: systemic arterial hypertension
USD: United States dollars
UTI: urinary tract infection
UUI: urinary urge-incontinence
WHO: World Health Organization
